# Naringenin Nanocrystals Containing Pluronic^®^ F127 Hydrogel for Skin Cancer Treatment

**DOI:** 10.3390/gels12040336

**Published:** 2026-04-17

**Authors:** Mayank Sharma, Neha Maheshwari, Rahul Maheshwari

**Affiliations:** 1 School of Pharmacy and Technology Management, SVKM’s Narsee Monjee Institute of Management Studies (NMIMS) Deemed-to-be-University, Shirpur 425405, Maharashtra, India; mayank2306@gmail.com; 2 School of Pharmacy and Technology Management, SVKM’s Narsee Monjee Institute of Management Studies (NMIMS) Deemed-to-be-University, Jadcherla, Hyderabad 509301, Telangana, India; nehamaheshwari0288@gmail.com

**Keywords:** Naringenin nanocrystals, Pluronic^®^ F127 hydrogel, solubility enhancement, ex vivo permeation, ROS-mediated apoptosis, skin delivery

## Abstract

Naringenin (NRG), a poorly water-soluble flavonoid with anticancer potential, suffers from limited bioavailability due to low aqueous solubility and poor membrane permeation. In this study, NRG nanocrystals (NRG-NCs) were developed using an optimized antisolvent precipitation–probe sonication method and incorporated into a 20% (*w*/*w*) Pluronic^®^ F127 hydrogel for enhanced delivery. The optimized NRG-NCs exhibited a mean particle size of ~195 ± 5 nm, polydispersity index of ~0.20 ± 0.02, and zeta potential of −24 ± 3 mV. Percentage yield and drug loading capacity were 88.6 ± 2.3% and 78.4 ± 1.8%, respectively. Nanocrystal formation resulted in ~9-fold enhancement in saturation solubility compared to raw NRG. The NRG-NCs gel demonstrated rapid dissolution (~90% release within 120 min) and ~2.5-fold higher ex vivo permeation across the Strat-M^®^ membrane relative to pure NRG. The hydrogel exhibited suitable physicochemical properties (viscosity ~12,850 cP; pH 6.2 ± 0.1; spreadability 5.8 ± 0.3 cm) and maintained >92% drug content after 30 days of refrigerated storage. Mechanistic studies revealed dose-dependent cytotoxicity, characterized by increased intracellular ROS, mitochondrial membrane depolarization, and elevated caspase-3 activity, confirming ROS-mediated apoptosis. In conclusion, the nanocrystal–hydrogel platform significantly enhances the solubility, permeation, and pro-apoptotic efficacy of NRG, demonstrating its potential for skin cancer treatment.

## 1. Introduction

A large proportion of bioactive phytoconstituents and small-molecule therapeutics exhibit limited water solubility, leading to poor dissolution, reduced membrane permeation, and suboptimal intracellular drug availability [1,2]. Naringenin (NRG), a naturally occurring flavonoid with documented antioxidant [3], anti-inflammatory [4], and anticancer properties [5,6], is a representative example of such compounds. Despite its promising therapeutic potential, the clinical translation of NRG has been severely restricted by its low aqueous solubility, poor dissolution kinetics, and limited cellular uptake [7].

Several formulation strategies have been explored to overcome solubility limitations, including polymeric nanoparticles [8,9], liposomes [10,11], solid dispersions [12], and inclusion complexes [13]. However, these systems often involve complex preparation procedures, low drug loading, stability challenges, or the use of high amounts of excipients. Nanocrystal technology has emerged as an attractive alternative for improving the bioavailability of poorly water-soluble drugs without altering their chemical structure [14,15]. Nanocrystallization enhances the apparent solubility and dissolution behavior of poorly water-soluble drugs without the need for cosolvents or chemical modification, primarily through physicochemical size reduction effects. When drug particles are reduced to the nanometer scale, their specific surface area increases dramatically, leading to an enhanced dissolution rate as described by the Noyes–Whitney equation [16]. In addition, according to the Ostwald–Freundlich equation, a reduction in particle size increases surface curvature, which elevates saturation solubility due to higher surface free energy. This phenomenon results in an increased dissolution pressure at the particle–liquid interface, thereby improving thermodynamic solubility [17]. Furthermore, nanocrystals exhibit reduced diffusion-layer thickness and improved wettability, especially in the presence of minimal stabilizers, thereby facilitating faster drug dissolution and enhanced concentration gradients across biological membranes. Unlike conventional approaches such as cosolvency or salt formation, nanocrystal technology preserves the drug’s chemical integrity while achieving high drug loading and improved bioavailability through purely physical modification. However, stabilization of nanocrystal is challenging and require special consideration.

In context, Octenyl succinic anhydride (OSA)-modified starch (OSS) has emerged as an effective stabilizer for nanocrystal systems due to its unique amphiphilic structure, comprising hydrophobic octenyl chains and hydrophilic polysaccharide backbones [18,19]. This dual functionality enables strong adsorption at the drug-water interface, providing steric stabilization and preventing nanocrystal aggregation. The hydrophobic domains of OSS interact with the poorly water-soluble drug surface, while the hydrophilic segments extend into the aqueous phase, forming a protective barrier that enhances dispersion stability and wettability. Such stabilization is particularly advantageous in nanocrystal systems, where high surface energy can otherwise promote particle agglomeration.

In addition, IR780 iodide, a near-infrared (NIR) fluorescent dye, has attracted significant attention in cancer theranostics for its combined photothermal, photodynamic, and imaging capabilities [20]. Upon NIR irradiation, IR780 can generate localized heat and reactive oxygen species (ROS), thereby enhancing cancer cell apoptosis [21]. Furthermore, its intrinsic fluorescence enables real-time tracking of cellular uptake and distribution [22]. Incorporation of IR780 into nanocrystal-based hydrogel systems may therefore provide a multifunctional platform that combines improved drug delivery with potential imaging and synergistic therapeutic effects.

Beyond solubility enhancement, localized and controlled drug delivery remains critical for improving therapeutic efficacy and minimizing systemic exposure [23]. Thermoresponsive hydrogels, such as Pluronic^®^ F127, offer a promising platform for sustained, site-specific drug delivery [24,25,26]. These systems exhibit sol–gel transition behavior, enabling ease of administration and prolonged residence at the target site [27]. Incorporating nanocrystals into a hydrogel matrix may combine the advantages of enhanced dissolution, sustained release, and improved tissue retention [28].

In addition to improving physicochemical performance, it is essential to elucidate the biological mechanism underlying enhanced therapeutic activity. Naringenin has been reported to induce apoptosis through oxidative stress-mediated pathways [29,30]. Increased intracellular reactive oxygen species (ROS) can disrupt mitochondrial membrane potential (MMP), activate caspase cascades, and ultimately trigger programmed cell death. Therefore, a formulation strategy that enhances intracellular delivery of NRG may potentiate its pro-apoptotic efficacy via mitochondrial signaling pathways.

**Figure 1:**
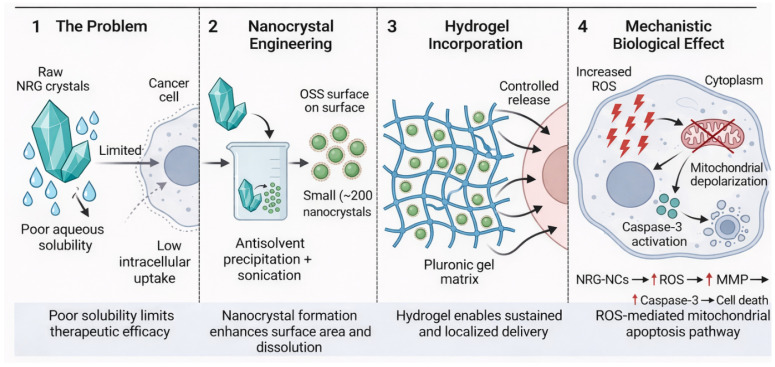
Schematic representation of the nanocrystal-hydrogel platform for enhanced naringenin delivery and ROS-mediated mitochondrial apoptosis. Raw NRG crystals exhibit poor aqueous solubility, limited permeation, and low intracellular uptake. Antisolvent precipitation combined with probe sonication yields OSS-stabilized NRG nanocrystals (~200 nm), which are subsequently incorporated into a Pluronic^®^ F127 hydrogel matrix for controlled, localized release. Enhanced intracellular delivery promotes ROS generation, mitochondrial membrane depolarization, caspase-3 activation, and apoptosis. Created with BioRender.com.

Based on these considerations, we hypothesized that engineering NRG into nanocrystals and subsequently incorporating them into a Pluronic^®^ F127 hydrogel could simultaneously enhance solubility, dissolution, permeation, and apoptotic activity. The overall conceptual framework of this strategy is illustrated in Figure 1, which depicts (i) the limitation of raw NRG crystals, (ii) nanocrystal engineering via antisolvent precipitation and sonication, (iii) hydrogel-mediated controlled delivery, and (iv) ROS-mediated mitochondrial apoptosis.

In the present study, NRG nanocrystals were developed using an optimized antisolvent precipitation–probe sonication approach and stabilized with an appropriate surfactant system. The optimized nanocrystals were subsequently incorporated into a thermoresponsive hydrogel matrix. Comprehensive physicochemical characterization, solubility and dissolution assessment, ex vivo permeation studies, and mechanistic biological evaluations, including ROS generation, mitochondrial membrane potential analysis, and caspase-3 activation, were performed to validate the proposed strategy. This integrated nanocrystal-hydrogel platform aims to provide a rational, scalable approach to enhance the therapeutic performance of poorly soluble flavonoids and may serve as a promising candidate for advanced localized anticancer drug-delivery applications.

## 2. Results and Discussion

### 2.1. Optimization and Physicochemical Characterization of NRG-NCs

The antisolvent precipitation method combined with probe sonication successfully produced OSS-stabilized NRG nanocrystals (NRG-NCs). The influence of critical formulation and process variables, including solvent-to-antisolvent ratio, stabilizer concentration, and sonication power, was systematically evaluated to obtain nanocrystals with minimal particle size and narrow size distribution.

**Figure 2:**
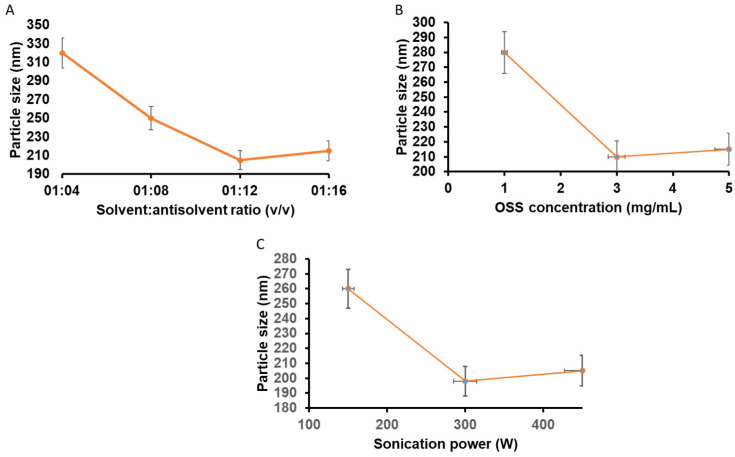
Optimization of NRG nanocrystals: effect of formulation and process parameters on particle size. (**A**) Influence of solvent-to-antisolvent ratio. (**B**) Effect of OSS stabilizer concentration. (**C**) Effect of sonication power. Data represent mean ± SD (n = 3).

As shown in Figure 2A, increasing the solvent-to-antisolvent ratio from 1:4 to 1:12 resulted in a significant reduction in hydrodynamic particle size. At a lower ratio (1:4), larger particles (~320 ± 15 nm) were obtained, likely due to insufficient instantaneous supersaturation, where crystal growth predominated over nucleation. Increasing the antisolvent volume enhanced supersaturation kinetics, promoting rapid burst nucleation consistent with LaMer’s model, thereby reducing particle size to ~205 ± 8 nm at a 1:12 ratio. A further increase to 1:16 did not produce additional size reduction and slightly increased particle size and PDI, possibly due to reduced collision efficiency and mild secondary aggregation at excessive dilution. These findings confirm that nucleation kinetics were strongly governed by supersaturation dynamics.

The effect of OSS concentration on particle size is illustrated in Figure 2B. At 1 mg/mL OSS, incomplete surface coverage of newly formed nanocrystals resulted in larger particles (~280 ± 12 nm) and broader distribution (PDI ≈ 0.38). Increasing OSS concentration to 3 mg/mL significantly reduced particle size (~210 ± 7 nm) and improved uniformity (PDI ≈ 0.22 ± 0.03), indicating optimal steric stabilization through efficient surface adsorption of OSS. Further increasing to 5 mg/mL did not yield additional size reduction and slightly increased particle size, possibly due to increased viscosity and diffusion resistance, which may have influenced the nucleation-growth balance. Therefore, 3 mg/mL OSS was selected as the optimal stabilizer concentration.

The influence of sonication power on nanocrystal size is presented in Figure 2C. At 150 W, particle size remained above 250 nm, suggesting insufficient acoustic cavitation to effectively disrupt loosely formed microcrystals. Increasing sonication power to 300 W significantly reduced particle size to approximately 198 ± 6 nm with a narrow PDI (~0.21 ± 0.02), reflecting efficient cavitation-induced fragmentation and deaggregation. However, further increase to 450 W did not significantly reduce particle size and caused slight broadening of distribution, potentially due to localized thermal effects and microstreaming-induced secondary aggregation. An optimal sonication duration of 6 min was identified, as prolonged exposure did not provide additional size reduction.

The optimized NRG-NC formulation exhibited a mean hydrodynamic diameter of approximately 195 ± 5 nm, a PDI of 0.20 ± 0.02, and a zeta potential of −24 ± 3 mV. The negative surface charge is attributed to ionized carboxyl groups of OSS, contributing to combined steric and electrostatic stabilization. Collectively, these findings demonstrate that particle size reduction was governed by the interplay between supersaturation-driven nucleation, stabilizer surface adsorption, and controlled acoustic energy input. The optimized formulation was selected for subsequent structural characterization and for incorporation into the hydrogel.

The optimization of formulation and process parameters was performed in a sequential manner to systematically evaluate their individual effects on nanocrystal characteristics. Initially, the solvent-to-antisolvent ratio was optimized, and the selected ratio (1:12 *v*/*v*) was subsequently used as a fixed parameter for evaluating the effect of OSS concentration. Following this, both optimized parameters were maintained constant while optimizing sonication power and duration. Based on these studies, the final optimized formulation parameters were identified as a solvent-to-antisolvent ratio of 1:12 (*v*/*v*), OSS concentration of 3 mg/mL, sonication power of 300 W, and sonication time of 6 min. These optimized conditions were used for all subsequent characterization and biological investigations.

### 2.2. % Yield and Drug Loading Capacity

The percentage yield and drug loading capacity of the optimized NRG nanocrystals (NRG-NCs) were determined to assess the efficiency of the antisolvent precipitation–sonication process and the stabilizing performance of OSS. The quantitative results are summarized in Table 1. The optimized formulation exhibited a percentage yield of ~88.6 ± 2.3%, indicating efficient recovery of nanocrystals following centrifugation and drying. The minor yield loss may be attributed to incomplete precipitation of extremely fine particles, drug adsorption to processing vessels, and removal of loosely bound drug during centrifugation. Nevertheless, the yield above 85% confirms that the selected solvent-to-antisolvent ratio and sonication conditions were suitable for scalable nanocrystal production. The optimized NRG-NCs exhibited a drug loading capacity (LC) of ~78.4 ± 1.8%, indicating a high drug content within the nanocrystal system. Since nanocrystals consist predominantly of pure drug stabilized by minimal amounts of surfactant, high loading values are expected compared to polymeric nanoparticle systems. The high LC confirms that OSS primarily functions as a surface stabilizer rather than a bulk carrier, preserving the intrinsic drug mass within the nanocrystals. The UV-visible spectrophotometric method used for quantification showed good linearity within the concentration range of 2–12 µg/mL, with a regression coefficient (R^2^) of 0.998–0.999, confirming the reliability of drug estimation. The calculated LOD and LOQ values (e.g., LOD ≈ 0.18 µg/mL; LOQ ≈ 0.55 µg/mL) indicated adequate sensitivity of the analytical method for low-concentration detection. Triplicate measurements demonstrated low variability, indicating acceptable reproducibility of both the formulation and the analytical procedures. Overall, the high yield and substantial drug-loading capacity confirm the efficiency of the nanocrystal fabrication process and validate OSS as an effective stabilizing agent. These findings support the suitability of the optimized formulation for further physicochemical and biological evaluation.

### 2.3. Structural and Morphological Characterization of NRG-NCs

**Figure 3:**
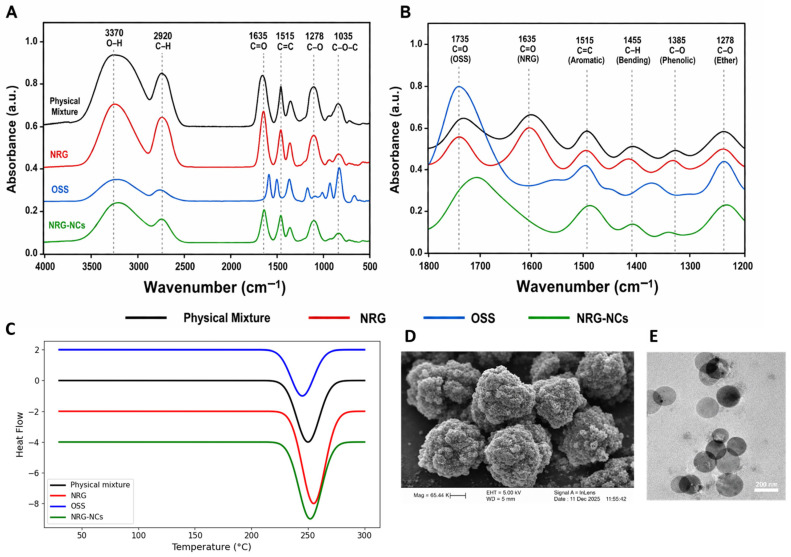
Structural characterization of NRG nanocrystals. (**A**) FTIR spectra (4000–500 cm^−1^) of raw NRG, OSS, physical mixture, and NRG-NCs showing characteristic functional group peaks. (**B**) Expanded FTIR fingerprint region (1800–1200 cm^−1^) highlighting peak shifts and interactions. (**C**) DSC thermograms illustrating thermal behavior and crystalline characteristics. (**D**) SEM micrograph showing surface morphology. (**E**) TEM image confirming nanoscale size and morphology.

The structural integrity, solid-state characteristics, and morphological features of the optimized NRG nanocrystals (NRG-NCs) were comprehensively evaluated using FTIR spectroscopy, differential scanning calorimetry (DSC), scanning electron microscopy (SEM), and transmission electron microscopy (TEM). The integrated results are presented in Figure 3A–E).

The FTIR spectra of raw NRG, OSS, physical mixture, and NRG-NCs are presented in Figure 3A,B. Raw NRG exhibited characteristic peaks corresponding to O–H stretching (~3200–3400 cm^−1^), C=O stretching (~1630–1650 cm^−1^), and aromatic C=C stretching (~1500–1600 cm^−1^). OSS showed prominent peaks associated with hydroxyl groups (~3300 cm^−1^) and C–O–C stretching vibrations (~1000–1150 cm^−1^). The physical mixture displayed a simple superimposition of peaks from both components without significant shifts, indicating the absence of chemical interaction. In contrast, NRG-NCs exhibited slight peak broadening and minor shifts in the O–H and C=O regions, suggesting weak intermolecular interactions such as hydrogen bonding between NRG and OSS. Importantly, no new peaks or disappearance of characteristic peaks were observed, confirming the preservation of NRG’s chemical integrity during nanocrystal formation.

DSC thermograms (Figure 3C) revealed a sharp endothermic peak of raw NRG at ~250–255 °C, corresponding to its crystalline melting point. OSS exhibited a broad endothermic transition indicative of its amorphous nature. The physical mixture retained the characteristic melting peak of NRG with a slight reduction in intensity. In the NRG-NCs formulation, the NRG melting endotherm was retained but showed reduced intensity and slight broadening, indicating a reduction in crystallite size rather than complete amorphization. This behavior is consistent with nanocrystal formation, in which reduced particle size leads to decreased lattice energy and partial disruption of long-range order, without altering the drug’s fundamental crystalline nature. The observed reduction in melting peak intensity and slight broadening in DSC thermograms suggests a decrease in crystal domain size rather than complete amorphization.

Morphological evaluation using SEM (Figure 3D) revealed that the optimized NRG-NCs consisted of discrete, near-spherical to slightly irregular particles with minimal aggregation. The observed particle dimensions were consistent with the hydrodynamic diameter obtained from DLS analysis (~195 ± 5 nm), confirming successful size reduction via the antisolvent precipitation–sonication process.

TEM analysis (Figure 3E) further confirmed the nanoscale dimensions and uniformity. The nanocrystals appeared electron-dense and well-dispersed, with particle sizes of ~180–200 nm. The slightly smaller size observed in TEM compared to DLS measurements can be attributed to the absence of the hydration layer and steric stabilization shell in dry-state imaging. The morphology corroborates effective stabilization by OSS and supports the narrow size distribution (PDI ~0.20).

Collectively, the combined FTIR, DSC, SEM, and TEM analyses confirm that the antisolvent precipitation approach successfully produced stable NRG nanocrystals without altering the drug’s chemical structure, while reducing crystal size to the nanoscale and partially modifying crystal domain dimensions. These structural and morphological findings validate the optimized formulation for subsequent incorporation into hydrogels and biological evaluation.

### 2.4. Saturation Solubility and In Vitro Dissolution Performance

**Figure 4:**
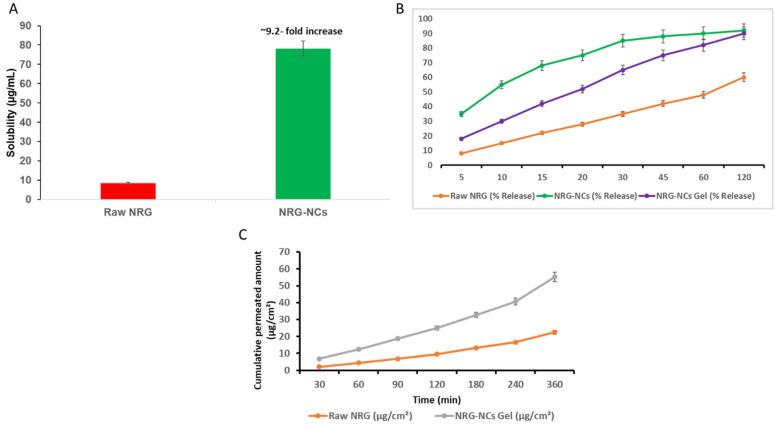
Enhanced physicochemical performance of NRG-NCs hydrogel. (**A**) Saturation solubility comparison between raw NRG and NRG-NCs. (**B**) In vitro dissolution profiles of raw NRG, NRG-NCs, and NRG-NCs hydrogel in PBS (pH 6.8) containing 0.2% Tween-80. (**C**) Ex vivo permeation profile across the Strat-M^®^ membrane comparing raw NRG and NRG-NCs hydrogel. Data are presented as mean ± SD (n = 3).

The impact of nanocrystal formation on the aqueous solubility of NRG was evaluated and is presented in Figure 4A. Raw NRG exhibited low intrinsic solubility (~8–9 µg/mL), consistent with its hydrophobic flavonoid structure. In contrast, the optimized NRG-NCs demonstrated a marked enhancement in saturation solubility (~78 µg/mL), corresponding to an approximately 9.2-fold increase compared to the pure drug. This significant solubility enhancement can be attributed to particle size reduction to the nanoscale (~195 nm), which increases surface area and elevates surface free energy. According to the Ostwald-Freundlich equation, a reduction in particle size increases dissolution pressure and thermodynamic solubility. Additionally, improved wetting and dispersion provided by OSS further contributed to enhanced aqueous interaction. The findings confirm that the antisolvent precipitation–sonication approach effectively improved the dissolution thermodynamics of NRG.

The dissolution profiles of raw NRG, NRG-NCs, and NRG-NCs incorporated hydrogel are shown in Figure 4B. Raw NRG exhibited slow and incomplete dissolution, reaching approximately 60% release at 120 min, reflecting its poor aqueous solubility and limited surface availability. In contrast, NRG-NCs showed a rapid initial release phase, achieving ~55% release within 10 min and nearly complete release (~90–95%) within 60–120 min. This rapid dissolution behaviour is attributed to reduced diffusion layer thickness, increased specific surface area, and improved wettability associated with nanocrystals. Incorporation of NRG-NCs into the Pluronic^®^ F127 hydrogel modulated the release profile, producing a more controlled and sustained release pattern. The NRG-NCs gel formulation exhibited intermediate kinetics, achieving approximately 90% release at 120 min, with a reduced burst effect compared to free nanocrystals. This controlled behavior is likely due to the polymeric network of Pluronic^®^ F127, which imposes diffusional resistance and regulates drug transport from the gel matrix. Conclusively, these findings demonstrate that nanocrystal formation significantly enhances the solubility and dissolution rate of NRG, while hydrogel incorporation enables controlled release. The combined system thus offers both improved bioavailability and localized sustained delivery, supporting its suitability for subsequent permeation and biological evaluation.

### 2.5. Ex Vivo Permeation Study Using Strat-M^®^ Membrane

The ex vivo permeation behaviour of raw NRG and the optimized NRG-NCs hydrogel formulation was evaluated using the Strat-M^®^ synthetic membrane, and the cumulative permeation profiles are presented in Figure 4C. Raw NRG exhibited limited membrane permeation throughout the study period. The cumulative permeated amount increased gradually with time, reaching approximately ~22 µg/cm^2^ at 360 min. This restricted transport is attributed to the poor aqueous solubility and slow dissolution rate of the crystalline drug, which limit the available concentration gradient across the membrane interface. In contrast, the NRG-NCs gel formulation demonstrated significantly enhanced permeation at all time points. The cumulative permeated amount increased progressively, reaching approximately ~55 µg/cm^2^ at 360 min, representing nearly a 2.5-fold enhancement compared to raw NRG. The permeation profile exhibited a near-linear increase over time, suggesting diffusion-controlled transport across the membrane. The improved permeation of the NRG-NCs gel can be attributed to multiple synergistic factors. First, nanocrystal formation increased the apparent solubility and dissolution rate of NRG, thereby enhancing its thermodynamic activity. According to Fick’s first law of diffusion, the permeation flux is directly proportional to the concentration gradient; thus, increased drug availability at the donor interface enhances membrane transport. Second, the reduced particle size (~195 nm) facilitates closer, more uniform contact with the membrane surface, thereby improving diffusion efficiency. Third, OSS stabilization enhances wettability and dispersion, further promoting effective diffusion. Additionally, incorporation of NRG-NCs into the Pluronic^®^ F127 hydrogel matrix provided a sustained release environment at the donor interface, preventing rapid depletion of drug concentration and maintaining a consistent diffusion gradient. The controlled release from the gel matrix likely contributed to the steady and sustained permeation observed over the experimental duration.

### 2.6. Evaluation of Pluronic^®^ F127 Hydrogel

The physicochemical properties of the optimized NRG-NCs-loaded Pluronic^®^ F127 hydrogel were evaluated to assess suitability for skin application. The results are summarized in Table 2.

The NRG-NCs hydrogel exhibited a viscosity of approximately 12,850 ± 320 cP at 35 ± 0.5 °C and 100 rpm. The viscosity value indicates formation of a semi-solid gel structure with adequate mechanical strength for administration via skin. The thermoresponsive nature of Pluronic^®^ F127 promotes micellar aggregation at elevated temperatures, leading to gel formation at near-physiological skin temperatures. The measured viscosity is within the acceptable range for topical gels, ensuring sufficient residence time at the application site while maintaining ease of spreadability. The absence of abrupt viscosity fluctuations during triplicate measurements confirms uniform dispersion of nanocrystals within the polymeric matrix and indicates that incorporation of NRG-NCs did not destabilize the gel network.

The pH of the optimized hydrogel was 6.2 ± 0.1, within the physiologically acceptable skin pH range (4.5–6.5). Maintaining pH compatibility is critical to minimize irritation and preserve skin barrier integrity. The slightly acidic formulation is advantageous for topical applications and indicates chemical compatibility between NRG-NCs and the Pluronic^®^ F127 matrix. The hydrogel exhibited a mean spread diameter of 5.8 ± 0.3 cm under the applied load, demonstrating satisfactory spreadability. Adequate spreadability is essential for uniform drug distribution across the application site and patient compliance. The moderate spread diameter observed suggests an optimal balance between viscosity and flow behavior, allowing smooth application without excessive runoff. In conclusion, the viscosity, pH, and spreadability results confirm that the incorporation of NRG nanocrystals into the Pluronic^®^ F127 hydrogel produced a stable, skin-compatible, and user-friendly formulation. Importantly, the nanocrystal loading did not adversely affect the rheological or physicochemical properties of the hydrogel. These findings support the suitability of the developed system for skin therapeutic applications.

### 2.7. Cell Level Evaluations: Cytotoxicity, ROS Generation, Mitochondrial Dysfunction, and Caspase-3 Activation

The mechanistic basis underlying the enhanced anticancer activity of the NRG-NCs gel was investigated through in vitro cytotoxicity (MTT), intracellular reactive oxygen species (ROS) generation, mitochondrial membrane potential (MMP) disruption, and caspase-3 activation assays.

#### 2.7.1. Cytotoxicity Assessment (MTT Assay)

**Figure 5:**
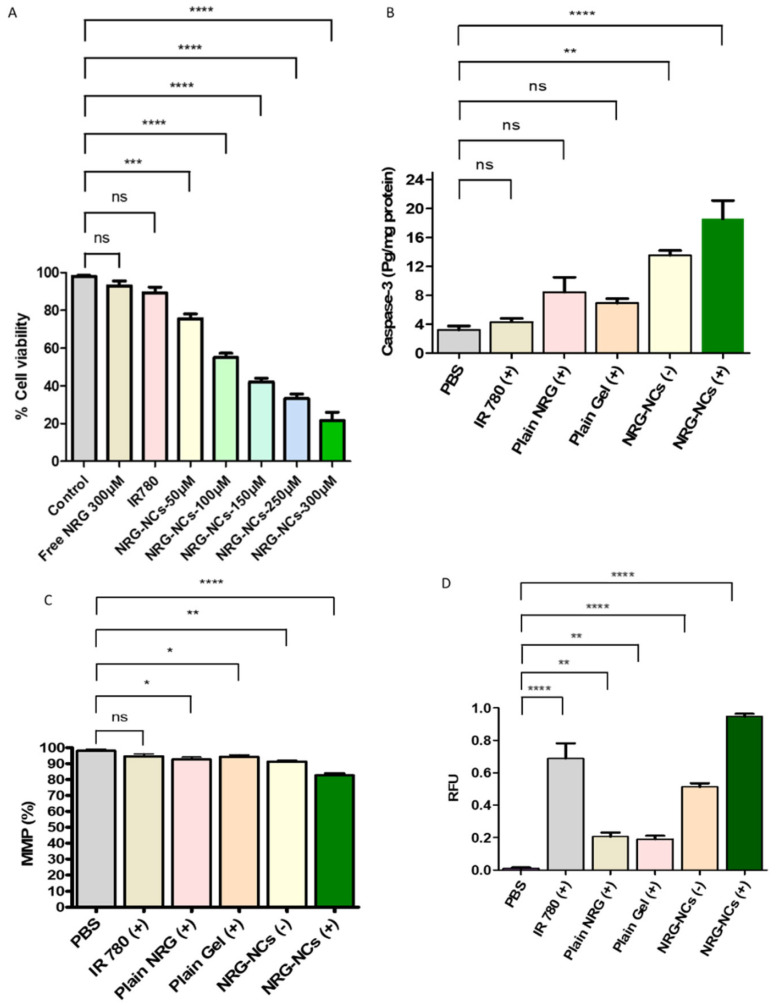
Mechanistic biological evaluation of NRG-NCs hydrogel. (**A**) MTT assay showing dose-dependent reduction in cell viability. (**B**) Caspase-3 activation confirming induction of apoptosis. (**C**) Mitochondrial membrane potential (MMP) assessment indicating mitochondrial depolarization (**D**) Intracellular ROS generation following treatment. Data represent mean ± SD (n = 3). Statistical significance indicated as * *p* < 0.05, ** *p* < 0.01, *** *p* < 0.001, **** *p* < 0.0001, ns: non-significant, versus control.

The MTT assay results (Figure 5A) demonstrated a clear dose-dependent reduction in cell viability following treatment with NRG-NCs gel. Control cells maintained approximately 100% viability, confirming assay validity. IR780-treated cells exhibited minimal cytotoxicity, indicating that the dye alone did not significantly affect cellular viability under the experimental conditions. Free NRG showed moderate cytotoxicity at higher concentrations; however, the NRG-NCs gel formulation induced significantly greater reduction in viability, particularly at elevated concentrations (*p* < 0.05). The enhanced cytotoxic effect of NRG-NCs gel compared to free NRG can be attributed to improved cellular uptake and increased intracellular availability resulting from nanoscale particle size and improved dispersion. These findings confirm that nanocrystal incorporation substantially enhances the biological efficacy of NRG. Recent studies consistently demonstrate that nanocarrier-based drug delivery systems significantly enhance intracellular drug accumulation and therapeutic efficacy compared to free drug counterparts. For instance, targeted nanocarriers facilitate receptor-mediated endocytosis, leading to improved cellular uptake and increased cytotoxicity in cancer cells [31]. Similarly, nanoparticle-based systems have been shown to enhance antiproliferative activity by increasing intracellular drug concentration and improving distribution within cancer cells [32]. Notably, IR780-based nanoformulations exhibit preferential mitochondrial accumulation and significantly lower IC_50_ values (2–4 fold reduction) compared to free drug, highlighting the advantage of nanoscale delivery systems [33]. These improvements are largely attributed to the unique physicochemical properties of nanoparticles, including optimized size, surface characteristics, and enhanced interaction with biological membranes, which collectively promote efficient cellular internalization and therapeutic response.

#### 2.7.2. Caspase-3 Activation

Caspase-3 activity, a hallmark of apoptosis execution, was assessed to confirm activation of downstream apoptotic pathways (Figure 5B). Cells treated with NRG-NCs gel exhibited significantly elevated caspase-3 activity relative to control and free NRG groups (*p* < 0.05). The increase in caspase-3 activity correlated with ROS elevation and mitochondrial depolarization, supporting activation of intrinsic apoptotic signaling. The coordinated increase in ROS, disruption of MMP, and activation of caspase-3 strongly indicate that the NRG-NCs gel induces apoptosis via the mitochondrial (intrinsic) pathway rather than necrotic cell death.

#### 2.7.3. Mitochondrial Membrane Potential (MMP) Disruption

The mitochondrial membrane potential, a key indicator of mitochondrial integrity and early apoptotic events, was evaluated using TMRM staining (Figure 5C). Control cells retained normal mitochondrial polarization, whereas treatment with NRG-NCs gel resulted in a significant loss of MMP in a dose-dependent manner. The reduction in mitochondrial membrane potential indicates mitochondrial dysfunction, which is commonly associated with ROS-induced apoptotic signaling. The NRG-NCs gel produced a more pronounced depolarization effect than free NRG, confirming enhanced pro-apoptotic activity via the mitochondrial pathway.

#### 2.7.4. Intracellular ROS Generation

To elucidate the role of oxidative stress in cytotoxicity, intracellular ROS levels were quantified (Figure 5D). Treatment with NRG-NCs gel resulted in a marked increase in ROS production compared to control and free NRG groups. The increase in ROS was concentration-dependent and significantly higher than that observed for IR780 alone. Elevated ROS generation is a well-established trigger of apoptosis via oxidative stress-mediated damage to cellular components. The amplified ROS levels observed in the NRG-NCs gel group suggest that nanocrystal-mediated enhancement of drug delivery promotes intracellular oxidative imbalance, thereby contributing to cytotoxicity.

### 2.8. Stability of NRG-NCs Hydrogel

**Figure 6:**
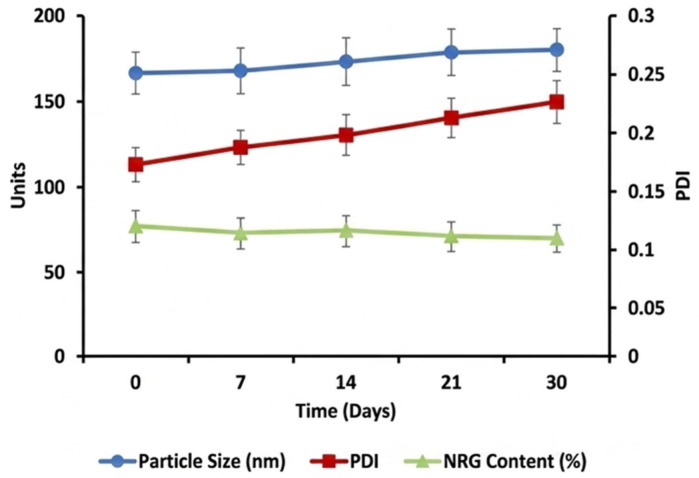
Storage stability of optimized NRG-NCs hydrogel under refrigerated conditions (4 ± 2 °C) for 30 days. Changes in particle size and drug content (%) over time. Data represent mean ± SD (n = 3).

The physical and chemical stability of the optimized NRG-NCs hydrogel formulation was evaluated over a 30-day storage period under refrigerated conditions (4 ± 2 °C). The variations in particle size, polydispersity index (PDI), and drug content are presented in Figure 6. The initial mean particle size of the optimized formulation was approximately ~195 nm. Over the 30-day storage period, a gradual, moderate increase in particle size was observed, reaching ~210 nm at day 30. This slight increase may be attributed to minimal aggregation or Ostwald ripening phenomena during storage. However, the particle size remained well below 250 nm, indicating that the nanocrystal system retained its nanoscale characteristics and colloidal stability throughout the study duration. The PDI values remained within an acceptable range (<0.30) over the storage period, suggesting that no significant broadening of particle size distribution occurred (inset fig). The absence of abrupt changes in PDI confirms that the OSS stabilizer effectively maintained dispersion uniformity and prevented extensive aggregation.

Drug content analysis revealed a minor reduction in NRG retention over time, with approximately ~92–93% of the initial drug content remaining after 30 days. The limited decline in drug content indicates satisfactory chemical stability of NRG within the nanocrystal-hydrogel system. The protective effect of the Pluronic^®^ F127 matrix, along with steric stabilization provided by OSS, likely contributed to minimizing degradation and preserving formulation integrity. Importantly, no visible phase separation, precipitation, or change in gel consistency was observed during storage. The combined physical and chemical stability results demonstrate that the NRG-NCs hydrogel formulation remains stable under refrigerated storage for at least 30 days. Overall, the stability findings confirm that the optimized nanocrystal-loaded hydrogel possesses adequate short-term storage stability, supporting its suitability for further preclinical and translational investigations.

## 3. Conclusions

In the present study, naringenin nanocrystals (NRG-NCs) were successfully prepared via antisolvent precipitation combined with probe sonication and subsequently incorporated into a Pluronic^®^ F127 hydrogel system. Systematic optimization of formulation and process parameters yielded nanocrystals with a mean particle size of ~195 nm, a narrow size distribution, and satisfactory colloidal stability. Structural characterization confirmed that nanocrystal formation did not induce chemical modification of NRG, while thermal analysis indicated a reduction in crystal domain size without complete amorphization. The nanosizing approach resulted in a substantial increase in saturation solubility (~9-fold) and a significantly improved dissolution rate compared to raw NRG-incorporation into the hydrogel matrix enabled controlled drug release while maintaining enhanced drug availability. Ex vivo permeation studies demonstrated markedly improved membrane transport of NRG-NCs gel relative to the pure drug, confirming that solubility and dissolution enhancement translated into improved diffusion behavior. Mechanistic biological evaluation revealed that the NRG-NCs gel induced significant cytotoxicity via a ROS-mediated mitochondrial apoptotic pathway, characterized by elevated intracellular ROS levels, loss of mitochondrial membrane potential, and caspase-3 activation. These findings confirm that nanocrystal-mediated delivery substantially potentiates the therapeutic activity of NRG. The optimized hydrogel formulation also exhibited satisfactory short-term storage stability over 30 days, with minimal changes in particle size, distribution, and drug content. Finally, the developed NRG-NCs hydrogel system integrates enhanced solubility, improved permeation, controlled release, and mechanistically validated biological activity. This nanocrystal-based platform represents a promising strategy for improving the therapeutic efficacy of poorly water-soluble flavonoids. It may serve as a potential candidate for advanced topical or localized anticancer delivery applications.

## 4. Materials and Methods

### 4.1. Experimental Materials

Naringenin (NRG, ≥98% purity), octenyl succinic anhydride (OSS) modified starch, Pluronic^®^ F127 (poloxamer 407), and IR780 iodide were procured from Sigma-Aldrich Inc. (St. Louis, MO, USA). Tween-80 (polysorbate 80), dimethyl sulfoxide (DMSO), potassium bromide (spectroscopic grade), uranyl acetate, and analytical grade ethanol were procured from Merck (Bengaluru, India). Phosphate-buffered saline (PBS) at pH 6.8 and 7.4 was prepared using analytical-grade reagents. Hydrochloric acid and sodium hydroxide used for pH adjustment were of analytical grade. Dulbecco’s Modified Eagle Medium (DMEM), fetal bovine serum (FBS), and penicillin–streptomycin solution were purchased from Gibco (Thermo Fisher Scientific, Waltham, MA, USA). Strat-M^®^ synthetic membranes were obtained from Merck Millipore (Rockville, MD, USA). All chemicals and solvents used were of analytical or cell culture grade and were used without further purification. Ultrapure water was obtained using a Milli-Q purification system (Direct Q3, Merck Life Science Pvt Ltd., Mumbai, India).

### 4.2. Preparation of OSS Stabilizer Solution

OSS solution was prepared as described before with slight modification [34]. Briefly, OSS was first dispersed in deionized water at different concentrations (1–5 mg/mL) under continuous magnetic stirring until complete hydration. The solution was then cooled to 4 °C before use in nanocrystal preparation to promote rapid nucleation during antisolvent precipitation.

### 4.3. Preparation of NRG NCs

Naringenin nanocrystals (NRG-NCs) were prepared using a bottom-up antisolvent precipitation technique [35] followed by probe sonication to control nucleation and crystal growth. Naringenin (16 mg) was accurately weighed and dissolved in 8 mL of absolute ethanol under gentle magnetic stirring at ambient temperature (25 ± 2 °C) until a clear solution was obtained. The solution was filtered through a 0.45 µm PTFE membrane filter to remove any undissolved particles and prevent heterogeneous nucleation during precipitation. OSS modified starch was dissolved in deionized water at concentrations ranging from 1 to 5 mg/mL. The stabilizer solution was stirred for complete hydration and subsequently cooled to below 4 °C using an ice bath for at least 15 min prior to precipitation. Cooling of the antisolvent phase was performed to enhance supersaturation rate and promote rapid nucleation over crystal growth. The organic phase was injected into the aqueous antisolvent phase under continuous magnetic stirring at 1200 rpm. Various solvent-to-antisolvent volume ratios (1:4, 1:8, 1:12, and 1:16 *v*/*v*) were investigated to evaluate their effect on particle formation. In a typical preparation, 3 mL of the ethanolic NRG solution was rapidly introduced into 12 mL of pre-cooled OSS solution. The addition was performed dropwise within 10–15 s to ensure rapid mixing and uniform supersaturation. Immediate turbidity indicated formation of nanosized drug crystals. The resulting nanosuspension was subjected to probe sonication (VC-505, Vibra Cell, Sonic, Hyderabad, India) to reduce particle size and minimize aggregation. Sonication parameters evaluated included: Power input: 150 W, 300 W, and 450 W and Duration: 3, 6, 9, and 12 min. Sonication was performed in pulse mode (5 s on/5 s off) to prevent thermal degradation. The sample container was maintained in an ice bath throughout processing, ensuring the temperature did not exceed 10 °C. Following sonication, the nanosuspension was centrifuged using a refrigerated high-speed centrifuge at 13,000 rpm for 25 min at 4 °C to separate free stabilizer and unprecipitated drug. The supernatant was carefully removed, and the pellet containing NRG-NCs was redispersed in cold deionized water. Redispersion was facilitated using a bath sonicator for 2–3 min to obtain a homogeneous nanosuspension.

### 4.4. Validation of NRG-NPs Using State-of-the-Art Techniques

Validation of NRG-NCs was performed using spectroscopic and thermal analytical techniques to confirm drug integrity, assess drug-stabilizer interactions, and evaluate potential changes in crystallinity following nanocrystal formation. UV-visible spectroscopic analysis was conducted using a pre-calibrated UV-1900i spectrophotometer (Shimadzu, Koriyama, Japan). Samples of pure NRG, OSS solution, and appropriately diluted NRG-NCs were prepared in ethanol to ensure complete drug solubilization before measurement. Baseline correction was performed using the corresponding solvent blank, and spectra were recorded in the range of 200–400 nm in spectrum mode. All measurements were carried out in triplicate to ensure reproducibility, and instrument stability was verified before analysis. Fourier transform infrared (FTIR) spectroscopy was employed to investigate potential intermolecular interactions between NRG and OSS in the nanocrystal formulation. Spectra of pure NRG, pure OSS, a physical mixture of NRG and OSS, and freeze-dried NRG-NCs were recorded using an Alpha-II FTIR spectrophotometer (Alpha-II, Bruker, Hyderabad, India). Each sample was thoroughly blended with spectroscopic-grade, dried potassium bromide (KBr) at approximately 1:100 (sample: KBr, *w*/*w*) and compressed into transparent pellets under hydraulic pressure. Spectra were collected over the wavenumber range of 4000–400 cm^−1^ with a resolution of 4 cm^−1^, and 32 scans were accumulated for each sample to improve signal-to-noise ratio. Characteristic functional group peaks were analyzed to detect possible hydrogen bonding, peak shifts, or changes in intensity indicative of molecular interactions. Thermal behavior and changes in crystallinity were examined using differential scanning calorimetry (DSC-6000, PerkinElmer, Waltham, MA, USA). Approximately 5 mg of each sample (NRG, OSS, physical mixture, and dried NRG-NCs) was accurately weighed and sealed in aluminum pans with pinhole lids to allow controlled vapor release. An empty sealed aluminum pan was used as a reference. Samples were heated from 20 °C to 240 °C at a constant rate of 10 °C/min under a continuous nitrogen purge (flow rate ~20 mL/min) to prevent oxidative degradation. Thermograms were analyzed to compare melting endotherms, peak shifts, enthalpy changes, and disappearance or reduction in crystalline peaks, thereby assessing possible amorphization or polymorphic transformation resulting from nanocrystal formation.

### 4.5. Size, Polydispersity Index, and Surface Electric Charge Determination

The hydrodynamic particle size, polydispersity index (PDI), and surface charge (zeta potential) of the prepared NRG nanocrystals (NRG-NCs) were determined using dynamic light scattering (DLS) and electrophoretic light scattering techniques (Zetasizer Nano ZS90, Malvern Instruments Ltd., Worcestershire, UK). Measurements were performed at 25 ± 0.1 °C using a fixed scattering angle of 170° (backscattering detection mode). Before analysis, freshly prepared nanosuspensions were diluted in filtered phosphate-buffered saline (PBS, pH 7.4) to minimize multiple scattering while maintaining colloidal stability. All samples were gently inverted to ensure uniform dispersion and were allowed to equilibrate thermally inside the instrument for at least 2 min before measurement. Disposable polystyrene cuvettes were used for particle size analysis, and folded capillary cells were used for zeta potential determination. Each sample was analyzed in triplicate, with at least three consecutive measurements per run, and the results were reported as mean ± standard deviation. PDI values below 0.3 were considered indicative of a narrow size distribution, while zeta potential values were interpreted to assess the electrostatic stability of the nanosuspension.

### 4.6. % Yield and Loading Content Determination

Percentage yield and drug loading capacity of the prepared NRG nanocrystals (NRG-NCs) were determined to evaluate process efficiency and the effectiveness of OSS as a stabilizing agent. After completion of antisolvent precipitation and probe sonication, the nanosuspension was centrifuged at 13,000 rpm for 25 min at 4 °C to separate nanocrystals from the aqueous supernatant containing unprecipitated or loosely bound drug. The supernatant was carefully collected and appropriately diluted with ethanol to ensure complete solubilization of free NRG before analysis. The concentration of unentrapped NRG in the supernatant was quantified using a UV-visible spectrophotometer (UV-1900i, Shimadzu, Koriyama, Japan) at 288 nm. The amount of NRG incorporated into nanocrystals was calculated by subtracting the free drug content in the supernatant from the total amount of NRG initially used in the formulation. The percentage yield of NRG-NCs was calculated using the following equation:

% Yield = (Weight of dried NRG-NCs recovered/Initial weight of NRG used) × 100(1)

Drug loading capacity (LC) was determined by dissolving a known quantity of centrifuged and redispersed NRG-NCs in ethanol to disrupt the nanocrystal structure and release the entrapped drug completely. The solution was diluted to the appropriate concentration and analyzed at 288 nm. Drug loading was calculated using Equation (2).

% LC = (Weight of NRG present in NRG-NCs/Total weight of NRG-NCs) × 100 (2)

For quantitative determination, a calibration curve of NRG was established in ethanol over a concentration range of 2–12 µg/mL. Standard solutions were prepared from a primary stock solution and analyzed at 288 nm in spectrum mode. Linearity was assessed by plotting absorbance versus concentration, and the regression equation was used for drug quantification. Method sensitivity was evaluated by calculating the limit of detection (LOD) and limit of quantification (LOQ) using the following Equation (3) and Equation (4), respectively:

LOD = 3.3 × (σ/S)(3)

LOQ = 10 × (σ/S)(4)

where σ represents the standard deviation of the response (peak absorbance of replicate blank or low-concentration samples), and S represents the slope of the calibration curve. All measurements were performed in triplicate, and results were expressed as mean ± standard deviation.

### 4.7. Microscopic Characterization

Surface morphology, particle shape, and size distribution of the prepared NRG nanocrystals (NRG-NCs) were examined using scanning electron microscopy (SEM), and transmission electron microscopy (TEM). For SEM analysis, nanocrystals were first lyophilized to obtain a dry powder and gently mounted onto aluminum stubs using double-sided conductive carbon tape. The samples were sputter-coated with a thin layer of gold (approximately 5–10 nm thick) for 30 s under an argon atmosphere to enhance surface conductivity and prevent charging. Imaging was performed using a Sigma 300 SEM (Zeiss, Oberkochen, Germany) operated under high vacuum at an accelerating voltage of 3.0 kV. Micrographs were captured at multiple magnifications to assess particle morphology and aggregation behavior.

For TEM analysis, a highly diluted nanosuspension was prepared using deionized water to avoid particle overlap. A drop (approximately 10 µL) of the diluted sample was placed onto a carbon-coated copper grid (200 mesh) and allowed to adsorb for 1–2 min. Excess liquid was carefully removed using filter paper, and the grid was negatively stained with 1–2% (*w*/*v*) aqueous uranyl acetate solution to enhance contrast. After air-drying at room temperature, samples were analyzed using a transmission electron microscope operated at an appropriate accelerating voltage (80–120 kV). Images were recorded to evaluate particle size, shape, and dispersion state.

### 4.8. Determination of Saturation Solubility

The equilibrium saturation solubility of pure NRG and optimized NRG nanocrystals (NRG-NCs) was determined in purified water at 37 ± 0.5 °C. An excess amount of each sample was added to 5 mL of water in tightly sealed glass vials to ensure the presence of undissolved drug throughout the experiment. The vials were placed in a thermostatically controlled shaking water bath maintained at 37 °C and agitated at 100 rpm for 48 h to achieve equilibrium. All samples were protected from light exposure to prevent potential photodegradation of NRG.

After equilibration, suspensions were centrifuged at 10,000 rpm (approximately 11,000–12,000× *g*) for 10 min at room temperature to separate undissolved particles. The supernatant was carefully collected and filtered through a 0.45 µm membrane filter to remove any residual particulate matter. The filtered solution was appropriately diluted with ethanol to ensure complete solubilization, and the diluted solution was analyzed spectrophotometrically at 288 nm using a previously validated calibration curve. All measurements were performed in triplicate, and results were expressed as mean ± standard deviation.

### 4.9. Preparation of Pluronic^®^ F127 Hydrogel and Loading of NRG-NPs

Pluronic^®^ F127 thermosensitive hydrogel was prepared using the cold method [36] to ensure complete polymer hydration and prevent premature gelation. Briefly, purified water was adjusted to pH 4.5 using dilute hydrochloric acid, and the solution was pre-cooled to 4 °C before polymer addition. Pluronic^®^ F127 powder was gradually added to the cold aqueous medium under continuous magnetic stirring to obtain a final polymer concentration of 20% *w*/*w*. The polymer was incorporated slowly in small portions to avoid lump formation and ensure uniform dispersion. Stirring was maintained at 4 °C until complete dissolution, and the dispersion was further stored at 4 °C for 12–24 h to allow complete hydration and formation of a clear, homogeneous solution.

For the preparation of NRG nanocrystal-loaded hydrogel, the optimized NRG-NCs suspension was pre-cooled to 4 °C and used as the aqueous phase. Pluronic^®^ F127 powder (20% *w*/*w* relative to total formulation weight) was gradually incorporated into the chilled nanocrystal suspension under continuous stirring at 4 °C. Polymer addition was performed slowly to prevent localized gelation and aggregation of nanocrystals. Stirring was continued until complete dissolution of the polymer was achieved, resulting in a uniform nanosuspension-based hydrogel. The final formulation was stored at 4 °C to maintain the sol state before further characterization and use.

### 4.10. Evaluation of Pluronic^®^ F127 Hydrogel

The physicochemical properties of the NRG nanocrystal-loaded Pluronic^®^ F127 hydrogel were evaluated for viscosity, pH, and spreadability. Viscosity was measured using a Brookfield DVE viscometer (Brookfield Engineering Laboratories, Middleboro, MA, USA) equipped with spindle no. 61 to 64. Approximately 25 g of hydrogel was placed in a cylindrical sample holder and equilibrated at 35 ± 0.5 °C for 10 min prior to measurement to simulate skin temperature. The measurement was performed at 100 rpm, and viscosity values were recorded once torque stabilization (variation < 1%) was achieved. Each sample was analyzed in triplicate (n = 3). For pH determination, 1 g of hydrogel was dispersed in 100 mL of distilled water, stirred for 5 min, and allowed to equilibrate. The pH was measured using a calibrated digital pH meter (Labmed, Gurgaon, India) with a glass electrode standardized using pH 4.0 and 7.0 buffer solutions. Measurements were performed in triplicate at room temperature. Spreadability was determined using a modified parallel plate method. A pre-weighed 1 g sample was placed between two glass plates (20 × 20 cm), and a standard weight of 100 g was applied for 30 s. The diameter of spread was measured along two perpendicular axes, and the mean value was calculated. Spreadability was also expressed using Equation (5):

S = (M × L)/T(5)

where M = applied weight (g), L = length spread (cm), and T = time (s). All measurements were conducted in triplicate. All determinations were conducted in triplicate.

### 4.11. In Vitro NRG Release from NRG-NPs Gel

The in vitro release behavior of NRG from the nanocrystal-loaded Pluronic^®^ F127 hydrogel was evaluated using a paddle-type dissolution apparatus under sink conditions. Phosphate-buffered saline (PBS, pH 6.8) containing 0.2% (*v*/*v*) Tween-80 was used as the dissolution medium to maintain sink conditions and enhance the wettability of the poorly water-soluble NRG. The dissolution study was performed at 37 ± 0.5 °C with a paddle rotation speed of 100 rpm. An amount of hydrogel equivalent to 2 mg of NRG was accurately weighed and introduced into 200 mL of dissolution medium. For comparison, dissolution studies were also conducted for pure NRG and optimized NRG nanocrystals (NRG-NCs) without gel incorporation under identical conditions. At predetermined time intervals (5, 10, 15, 20, 30, 45, 60, and 120 min), 1 mL of dissolution medium was withdrawn and immediately replaced with an equal volume of fresh pre-warmed medium to maintain constant volume and concentration gradient. Samples were filtered through a 0.22 µm membrane filter to remove any undissolved particles and diluted appropriately, if required, before analysis. The concentration of released NRG was quantified spectrophotometrically at 288 nm using a previously validated calibration curve. Cumulative percentage drug release was calculated, with dilution correction applied. All experiments were performed in triplicate, and results were expressed as mean ± standard deviation.

### 4.12. Ex Vivo Permeation Study Using Strat M^®^

The ex vivo permeation of NRG from the nanocrystal-loaded Pluronic^®^ F127 hydrogel was evaluated using Strat-M^®^ synthetic membrane, as performed by our group earlier [37]. Strat-M^®^ synthetic membrane structurally mimics human skin barrier properties [38]. The study was performed using vertical Franz diffusion cells with an effective diffusion area of approximately 1.77 cm^2^ and a receptor compartment volume of 10 mL. Before mounting, the Strat-M^®^ membrane was carefully hydrated in phosphate-buffered saline (PBS, pH 7.4) for 30 min to ensure proper equilibration. The membrane was then positioned between the donor and receptor compartments, with the shiny side facing the donor. The receptor compartment was filled with PBS (pH 7.4) containing 0.2% Tween-80 to maintain sink conditions for NRG, and the mixture was continuously stirred at 200 rpm using a magnetic stir bar. The system temperature was maintained at 32 ± 0.5 °C to simulate skin surface temperature (or 37 ± 0.5 °C if used), and care was taken to avoid air bubbles beneath the membrane. An accurately weighed amount of formulation equivalent to 200 µg of NRG (plain NRG dispersion and NRG-NCs hydrogel) was placed in the donor compartment. At predetermined intervals (0.5, 1, 2, 3, 4, and 6 h), 1 mL samples were withdrawn from the receptor compartment and immediately replaced with fresh pre-warmed medium. Samples were filtered through a 0.22 µm membrane filter and analyzed at 288 nm using a UV–Vis spectrophotometer. 

The withdrawn samples were filtered through a 0.22 µm membrane filter and analyzed spectrophotometrically at 288 nm using a validated calibration curve. The cumulative amount of drug permeated per unit area (µg/cm^2^) was calculated, and steady-state flux (J, µg/cm^2^/h) was determined from the slope of the linear portion of the permeation curve. The permeability coefficient (Kp) was calculated using the relation as shown in Equation (6):

Kp = J/C_0_ (6)

where C_0_ represents the initial drug concentration in the donor compartment. All experiments were conducted in triplicate, and results were expressed as mean ± standard deviation.

### 4.13. Cell Culture Evaluations

SK-MEL-28 cells were obtained from the National Centre for Cell Sciences (NCCS), Pune, India, and cultured in Dulbecco’s Modified Eagle Medium (DMEM) supplemented with 10% (*v*/*v*) fetal bovine serum (FBS) and 1% (*v*/*v*) penicillin–streptomycin solution. Cells were maintained in a humidified incubator at 37 °C with 5% CO_2_ atmosphere and subcultured upon reaching approximately 80–90% confluency.

#### 4.13.1. Cytotoxicity Test

The cytocompatibility of the NRG nanocrystal-loaded hydrogel was evaluated using the MTT assay [39]. SK-MEL-28 cells were seeded at 1 × 10^4^ cells per well in 96-well plates and allowed to attach overnight. Following attachment, cells were treated with various concentrations of NRG-NCs hydrogel equivalent to 50, 150, 250, and 350 µM NRG. Untreated cells served as the negative control. Additional comparison groups included cells treated with free NRG (350 µM) and IR780 dye at an optimized non-toxic concentration (5 µg/mL). For photothermal evaluation, selected groups were exposed to near-infrared (NIR) laser irradiation (808 nm) at a power density of 45 mW/cm^2^ for 10 min following 20 h of incubation with the formulations. Care was taken to maintain uniform irradiation distance and prevent excessive heating of the culture medium. After treatment, 10 µL of MTT solution (5 mg/mL in PBS) was added to each well and incubated for an additional 4 h at 37 °C to allow formation of formazan crystals. The supernatant was carefully removed, and the formed crystals were dissolved in 100 µL of dimethyl sulfoxide (DMSO). Absorbance was measured at 570 nm (reference wavelength 630 nm, if applicable) using a multimode microplate reader (Varioskan LUX, Thermo Fisher Scientific, Waltham, MA, USA). Cell viability was calculated using the following Equation (7):

% Cell viability = [(A_sample − A_blank)/(A_control − A_blank)] × 100(7)

where A_sample represents the absorbance of treated cells, A_control corresponds to untreated cells, and A_blank represents medium without cells. All experiments were performed in triplicate, and results were expressed as mean ± standard deviation.

#### 4.13.2. Mitochondrial Membrane Potential (MMP) Assay

MMP was evaluated using the tetramethylrhodamine methyl ester (TMRM) fluorescent probe, which selectively accumulates in active mitochondria in a membrane potential-dependent manner [40]. SK-MEL-28 cells were seeded in 96-well plates at a density of 5 × 10^3^ cells per well and allowed to adhere overnight under standard culture conditions (37 °C, 5% CO_2_). Cells were subsequently treated with the respective formulations (control and experimental groups) with or without near-infrared (NIR; 808 nm, 45 mW/cm^2^, 10 min) irradiation as previously described. After 20 h of incubation with the formulations, designated groups were irradiated with an 808 nm laser at a power density of 45 mW/cm^2^ for 10 min, followed by continued incubation to complete 24 h. Following treatment, cells were washed gently with phosphate-buffered saline (PBS) and incubated with TMRM solution (150 nM in serum-free medium) for 30 min at 37 °C in the dark to prevent photobleaching. After incubation, excess dye was removed by washing with PBS, and cells were resuspended in live-cell imaging buffer. MMP levels were quantified by measuring TMRM fluorescence intensity using flow cytometry, with excitation/emission settings appropriate for TMRM (Ex 548 nm/Em 573 nm). A decrease in fluorescence intensity relative to untreated control cells was interpreted as mitochondrial depolarization. All experiments were performed in triplicate, and data were expressed as mean ± standard deviation.

#### 4.13.3. Caspase-3 Assay

Caspase-3 activity was determined to evaluate apoptosis induction following treatment with the NRG nanocrystal-loaded hydrogel under normal and NIR-irradiated conditions. SK-MEL-28 cells were seeded in 96-well plates at a density of 5 × 10^3^ cells per well and allowed to adhere overnight under standard culture conditions (37 °C, 5% CO_2_). Cells were then treated with the designated formulations (control and experimental groups) with or without near-infrared (NIR) irradiation. After 20 h of incubation with the formulations, the designated NIR groups were irradiated with an 808 nm laser at a power density of 45 mW/cm^2^ for 10 min, followed by a further incubation to complete a total treatment duration of 24 h. Following treatment, cells were washed with cold phosphate-buffered saline (PBS) and lysed using the lysis buffer provided in the caspase-3 assay kit. The lysates were incubated on ice for 10–15 min and centrifuged at 10,000 rpm for 10 min at 4 °C to remove cellular debris. The supernatant containing total protein was collected, and protein concentration was determined (if performed; e.g., BCA assay) to allow normalization of caspase activity. Caspase-3 activity was measured using a colorimetric assay based on cleavage of a specific chromogenic substrate (e.g., Ac-DEVD-pNA), following the manufacturer’s protocol. Briefly, equal amounts of protein from each sample were incubated with the caspase-3 substrate at 37 °C for 2 h. Briefly 50 µL of protein extract was incubated with 50 µL reaction buffer containing 10 mM DTT and caspase-3 substrate (Ac-DEVD-pNA) at 37 °C for 1 h. Cleavage of the substrate released p-nitroaniline (pNA), and absorbance was measured at 405 nm using a microplate reader. Caspase-3 activity was calculated relative to untreated control cells and expressed as fold increase or percentage change. All experiments were performed in triplicate, and data were reported as mean ± standard deviation.

#### 4.13.4. Intracellular Reactive Oxygen Species (ROS) Determination

ROS generation was quantified to assess the photothermal and photodynamic effects of IR780 under NIR irradiation. SK-MEL-28 cells were seeded at a density of 5 × 10^3^ cells per well in 96-well plates and allowed to adhere overnight under standard culture conditions (37 °C, 5% CO_2_). Cells were then treated with the respective formulations (control and experimental groups) with or without near-infrared (NIR) exposure. After 20 h of incubation with the formulations, designated groups were irradiated with an 808 nm laser at a power density of 45 mW/cm^2^ for 10 min, followed by continued incubation to complete a total treatment duration of 24 h. Intracellular ROS levels were measured using 2′,7′-dichlorodihydrofluorescein diacetate (DCFH-DA), a cell-permeable fluorogenic probe [41]. Following treatment, cells were washed with phosphate-buffered saline (PBS) and incubated with DCFH-DA (10 µM in serum-free medium) for 30 min at 37 °C in the dark to prevent photo-oxidation. DCFH-DA diffuses into cells and is hydrolyzed by intracellular esterases to non-fluorescent DCFH, which ROS subsequently oxidizes to form highly fluorescent dichlorofluorescein (DCF). After incubation, excess dye was removed by washing with PBS, and fresh phenol red-free medium was added before measurement. Fluorescence intensity was measured using a microplate reader at approximately 485/525 nm excitation/emission wavelengths. Relative ROS levels were calculated by normalizing fluorescence intensity to untreated control cells. All experiments were conducted in triplicate, and data were expressed as mean ± standard deviation.

### 4.14. Stability Study of NRG-NCs Loaded Hydrogel

The short-term stability of the NRG nanocrystal-loaded Pluronic^®^ F127 hydrogel was evaluated under controlled storage and photostability conditions to assess its physicochemical integrity and drug retention over time. For storage stability, freshly prepared hydrogel samples were stored in sealed glass containers at 4 ± 1 °C (refrigerated) and 25 ± 2 °C (room temperature) for 30 days. At predetermined intervals (0, 15, and 30 days), samples were withdrawn and analyzed for changes in particle size, polydispersity index (PDI), and drug content. Particle size and PDI were measured using dynamic light scattering as previously described. Drug content was determined by dissolving a known quantity of hydrogel in ethanol, followed by spectrophotometric analysis at 288 nm using a validated calibration curve. The retention percentage of NRG was calculated using the following Equation (8):

% Retention = (NRG_t_/NRG_0_) × 100 (8)

where NRG_t_ represents the drug concentration at a given time point, and NRG_0_ represents the initial drug concentration at day 0. All experiments were conducted in triplicate, and results were expressed as mean ± standard deviation.

### 4.15. Statistical Analysis

All experimental data were expressed as mean ± standard deviation (SD) of at least three independent experiments (n = 3). Statistical analysis was performed using GraphPad Prism software (Version 10; GraphPad Software, San Diego, CA, USA). Differences between multiple groups were analyzed using one-way analysis of variance (ANOVA) followed by Tukey’s post hoc test for multiple comparisons. For comparisons between two groups, an unpaired Student’s *t*-test was applied where appropriate. A *p*-value of less than 0.05 was considered statistically significant. Statistical significance levels were indicated as follows: * *p* < 0.05, ** *p* < 0.01, and *** *p* < 0.001. All graphical data were generated using GraphPad Prism version 10, and curve fitting was performed using nonlinear regression analysis within the same software.

## Figures and Tables

**Table 1:** Percentage yield and drug loading capacity of the optimized NRG nanocrystals (NRG-NCs) along with validation parameters of the UV–visible spectrophotometric method. Values are expressed as mean ± SD (n = 3).

Parameters	Value (Mean ± SD, n = 3)
Percentage yield (%)	88.6 ± 2.3
Drug loading capacity (%)	78.4 ± 1.8
Regression coefficient (R^2^)	0.998
LOD (µg/mL)	0.18
LOQ (µg/mL)	0.55

**Table 2:** Physicochemical properties of optimized NRG-NCs hydrogel.

Parameters	Value (Mean ± SD, n = 3)
Viscosity (cP)	12,850 ± 320
pH	6.2 ± 0.1
Spreadability (cm)	5.8 ± 0.3

## Data Availability

The raw data supporting the conclusions of this article will be made available by the authors on request.
